# Inhibition of CC chemokine receptor 1 ameliorates osteoarthritis in mouse by activating PPAR-γ

**DOI:** 10.1186/s10020-024-00823-w

**Published:** 2024-06-03

**Authors:** Hanqing Xu, Sheng Chen, Cheng Meng, Yi He, Xiao-jian Huang, Hong-bo You

**Affiliations:** Department of Orthopedics, Tongji Hospital, Tongji Medical College, Huazhong University of Science and Technology, 1095 Jiefang Avenue, Wuhan, Hubei China

**Keywords:** CCR1, Osteoarthritis, Cartilage, DMM

## Abstract

**Background:**

Osteoarthritis (OA) is a degenerative joint disease characterized by cartilage destruction and inflammation. CC chemokine receptor 1 (CCR1), a member of the chemokine family and its receptor family, plays a role in the autoimmune response. The impact of BX471, a specific small molecule inhibitor of CCR1, on CCR1 expression in cartilage and its effects on OA remain underexplored.

**Methods:**

This study used immunohistochemistry (IHC) to assess CCR1 expression in IL-1β-induced mouse chondrocytes and a medial meniscus mouse model of destabilization of the medial meniscus (DMM). Chondrocytes treated with varying concentrations of BX471 for 24 h were subjected to IL-1β (10 ng/ml) treatment. The levels of the aging-related genes P16INK4a and P21CIP1 were analyzed via western blotting, and senescence-associated β-galactosidase (SA-β-gal) activity was measured. The expression levels of inducible nitric oxide synthase (iNOS), cyclooxygenase-2 (COX-2), aggrecan (AGG), and the transcription factor SOX9 were determined through western blotting and RT‒qPCR. Collagen II, matrix metalloproteinase 13 (MMP13), and peroxisome proliferator-activated receptor (PPAR)-γ expression was analyzed via western blot, RT‒qPCR, and immunofluorescence. The impact of BX471 on inflammatory metabolism-related proteins under PPAR-γ inhibition conditions (using GW-9662) was examined through western blotting. The expression of MAPK signaling pathway-related molecules was assessed through western blotting. In vivo, various concentrations of BX471 or an equivalent medium were injected into DMM model joints. Cartilage destruction was evaluated through Safranin O/Fast green and hematoxylin–eosin (H&E) staining.

**Results:**

This study revealed that inhibiting CCR1 mitigates IL-1β-induced aging, downregulates the expression of iNOS, COX-2, and MMP13, and alleviates the IL-1β-induced decrease in anabolic indices. Mechanistically, the MAPK signaling pathway and PPAR-γ may be involved in inhibiting the protective effect of CCR1 on chondrocytes. In vivo, BX471 protected cartilage in a DMM model.

**Conclusion:**

This study demonstrated the expression of CCR1 in chondrocytes. Inhibiting CCR1 reduced the inflammatory response, alleviated cartilage aging, and retarded degeneration through the MAPK signaling pathway and PPAR-γ, suggesting its potential therapeutic value for OA.

**Graphical Abstract:**

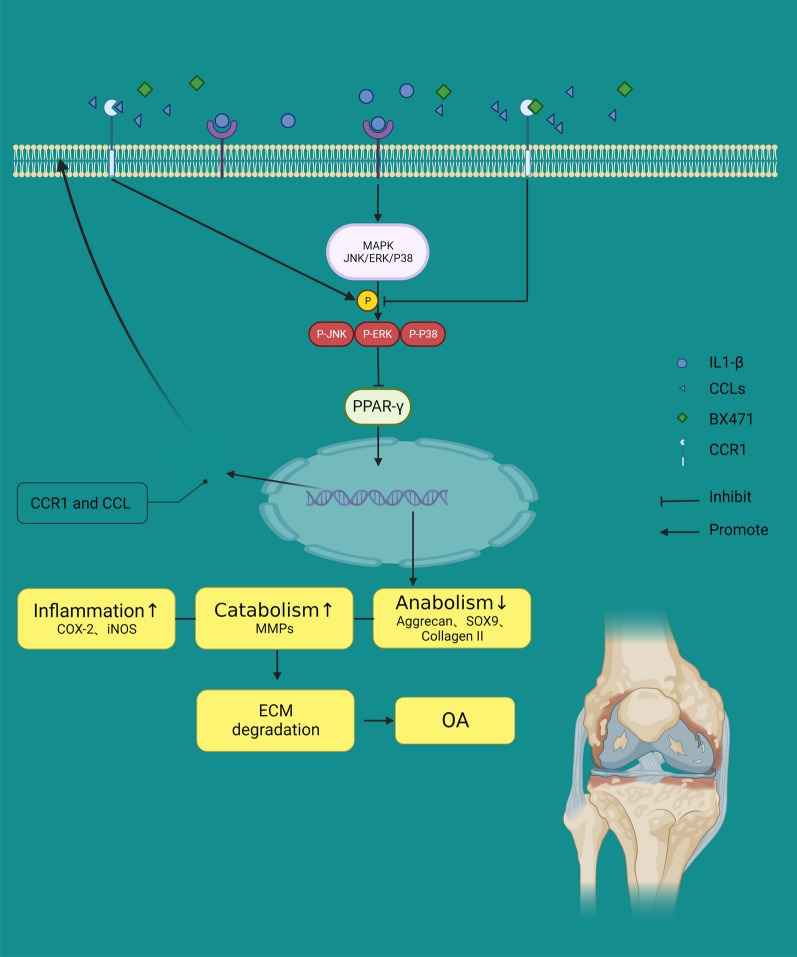

**Supplementary Information:**

The online version contains supplementary material available at 10.1186/s10020-024-00823-w.

## Introduction

Osteoarthritis (OA) is the most prevalent form of joint disease, affecting a significant portion of the global population, particularly the elderly population (Acklin et al. 2016). Its primary characteristics include articular cartilage degeneration, subchondral osteosclerosis, osteophyte formation, and synovial hyperplasia (Glyn-Jones et al. 2015). Individuals with OA commonly experience joint swelling, pain, and other clinical symptoms. As the disease progresses, a substantial number of patients require joint replacement to alleviate pain and prevent disability (Glyn-Jones et al. 2015). Despite its prevalence, the specific etiology and mechanisms underlying OA remain elusive. Notably, cartilage injury plays a pivotal role in OA development, including aging-related injury of chondrocytes and alterations in the extracellular matrix (Zheng et al. 2021). Inflammation has also emerged as a prominent feature of OA (Cho et al. 2015), with inflammatory factors such as TNF-α and IL-1β (Cho et al. 2015) showing elevated levels in the synovium, cartilage, and synovial fluid of OA patients. Numerous studies have indicated that these inflammatory factors stimulate the secretion of matrix metalloproteinases (MMPs) (Li et al. 2020; Kapoor et al. 2011; Kobayashi et al. 2005), iNOS, and COX-2, exacerbating chondrocyte injury (Cho et al. 2015) and amplifying the degradation of the extracellular matrix, including collagen II and AGG (Li et al. 2020). Although several treatments are available (Glyn-Jones et al. 2015), few have been shown to be effective, ultimately leading many patients to resort to joint replacement surgery.

Chemokines, a class of small molecule secreted proteins that bind to seven-transmembrane G protein-coupled receptors (Haringman et al. 2006; Vorst et al. 2015), are implicated in the inflammatory response of white blood cells, guiding cell migration and recruiting inflammatory cells from peripheral blood to inflammatory tissues. Over 50 chemokines and 20 chemokine receptors have been identified to date (Vorst et al. 2015) and are classified into the CXC, CC, C, and CX3C supergene families, with corresponding receptors named CXCR, CCR, CR, and CX3CR and ligands designated CXCL, CCL, CL, and CX3CL (Haas et al. 2005). In addition to their recruitment function, chemokines and their receptors regulate vascularization, stimulate mediator release, play a core role in inflammatory responses, and widely participate in organ development, immune surveillance, tissue regeneration, and other processes (Vorst et al. 2015; Mughees et al. 2022; Legler and Thelen 2016). Targeting chemokines and their receptors is considered a potential treatment for various immunoinflammatory diseases (Vorst et al. 2015). Studies have demonstrated that blocking CCR1 can inhibit macrophage infiltration in the synovium of knee joints in rheumatoid arthritis (RA) patients (Tak et al. 2013). However, the expression and function of CCR1 in chondrocytes remain controversial (Kholodnyuk et al. 2019; Silvestri et al. 2003), and its involvement in the onset and progression of OA remains unclear. Inhibiting its activity with small molecule inhibitors, such as BX471 (ZK-811752), a highly selective CCR1 antagonist, may represent a potential avenue for OA treatment, as it is 250 times more selective than CCR2, CCR5, and CXCR4 (Horuk 2005).

This study aimed to elucidate the expression of CCR1 in chondrocytes, analyze its impact on the inflammatory response, anabolism, and aging of mouse chondrocytes, and explore the potential underlying mechanisms involved. Additionally, we assessed the destabilization of the medial meniscus (DMM) in mice and the outcomes of mice following CCR1 blockade in the DMM group.

## Materials and methods

### Chemicals and reagents

The CCR1 inhibitor BX471 (ZK-811752) was obtained from MedChem Express (MCE). The PPAR-γ inhibitor GW9662 (HY-16578) was obtained from MedChem Express (MCE). Recombinant mouse IL-1β was obtained from R&D Systems (501-RL-010, United States). The primary antibodies used in this study were anti-CCR1, anti-Aggrecan, which were acquired from ABclonal Technology (Wuhan, China); anti-PPAR-γ, anti-p16INK4A, and anti-p21CIP1, which were acquired from Abcam (Shanghai, China); anti-iNOS, anti-MMP13, anti-Collagen II, anti-SOX9, anti-COX-2, anti-phospho-P38, anti-phospho-ERK 1/2, and anti-phospho-JNK, which were purchased from Cell Signaling Technology (CST, Beverly, MA, USA); and anti-P38, anti-ERK 1/2, anti-JNK, and anti-GAPDH, which were supplied from Proteintech Group (Wuhan, China). The secondary antibodies collagenase type II, trypsin and fluorescein isothiocyanate (FITC) and cyanine-3 (CY-3) conjugated to pure goat anti-rabbit IgG were obtained from Boster Biological Technology (Wuhan, Hubei, China).

### Chondrocyte isolation and culture

Chondrocytes were isolated from the epiphyseal cartilage of the knee joint of 5-day-old C57BL/6J mice purchased from Bainter Biotechnology Co., Ltd. (Hubei, China). After that, the cartilage pieces were broken into powdered pieces with scissors. The cartilage fragments were digested with 0.25% trypsin for 30 min and centrifuged at 200×*g* for 5 min. Subsequently, 0.2% collagenase II was used to digest the tissue for 5–7 h at 37 °C. Finally, after centrifugation at 200×*g* for 5 min, a chondrocyte suspension was prepared by adding complete culture medium containing DMEM/F12 culture medium, 10% fetal bovine serum (FBS-S011021, NEWZERUM, Christchurch, New Zealand) and 1% penicillin/streptomycin, and the suspensions were uniformly planted in culture dishes at 37 °C with 5% CO2. When the cell density reached 80%, passaging and treatment were carried out. The second/third passages were selected for subsequent experiments.

### Cell viability assay

Cell viability was evaluated by using a CCK8 kit (TargetMol Chemicals Inc., Boston, US). Chondrocytes were seeded in 96-well plates at a density of 5 × 10^3^ per well. After cell attachment, the cells were stimulated with different concentrations of BX471 (2.5, 5 and 10 μM) with or without IL-1β (10 ng/ml) for 24 h. A mixture of 10 μl of CCK-8 solution and 100 μl of DMEM/F12 culture medium was added to each well. After 1 h of incubation, the viability of the chondrocytes was measured by a microplate reader (Bio-Rad, Richmond, CA, USA) at 450 mm.

### Immunofluorescence

Cells were seeded on 24-well plates at a density of approximately 2 × 10^4^ per well. After stimulation with IL-1β (10 ng/ml) with or without BX471 (10 μM), the chondrocytes were fixed in 4% paraformaldehyde for 15 min. Afterward, 0.2% Triton X-100 was used to cover the cells for 10 min, and the cells were blocked with 1% bovine serum albumin (BSA) for 30 min at room temperature (15–25 °C). After blocking, the cells were incubated with primary antibodies against collagen II (1:200 dilution), PPAR-γ (1:200 dilution) and MMP13 (1:200 dilution) at 4 °C overnight. After washing with phosphate-buffered saline (PBS) three times, the chondrocytes were incubated with FITC- or CY-3-conjugated pure goat anti-rabbit secondary antibody in the dark for 1 h at room temperature (15–25 °C). Finally, after staining with DAPI for 10 min, the cells were observed, and photographs were obtained with a fluorescence microscope.

### Western blotting

Chondrocytes subjected to different interventions were lysed in RIPA lysis buffer (Boster, Wuhan, China) supplemented with 1% protease and phosphatase inhibitors for 30 min on ice. The lysate was collected, and the cells were further disrupted by 20% intensity ultrasound. After centrifugation at 1600×*g* for 30 min at 4 °C, the protein concentration of the supernatant was measured by a BCA kit (Boster, Wuhan, China) with a microplate reader at a wavelength of 560 nm. Subsequently, protein markers and equivalent quality sample proteins were subjected to 10% or 12.5% sodium dodecyl sulfate‒polyacrylamide gel electrophoresis (SDS‒PAGE) and transferred to PVDF membranes (Millipore, Billerica, MA). The membranes were incubated with primary antibodies overnight at 4 °C after 1 h of blocking with 5% BSA. After being washed with Tris-buffered saline containing Tween (TBST) three times, the membranes were incubated with secondary antibodies for 1–2 h at room temperature. With the use of enhanced chemiluminescence reagent (Abbkine, California, USA), the purified protein bands were visualized, and images were acquired with a Bio-Rad scanner (Bio-Rad, USA). GAPDH served as the housekeeping gene. ImageJ software was used to analyze the bands.

### RNA extraction and RT‒qPCR

Indicators related to the synthesis, catabolism, and inflammation of chondrocytes at the gene level were evaluated by RT‒qPCR analysis. Total RNA from different groups of chondrocytes was extracted and purified with an RNA extraction kit (Omega Biotek, USA). The total RNA concentration of the samples was calculated by a microplate reader. Afterward, the RNA samples were reverse transcribed to cDNA utilizing cDNA synthesis (Vazyme, Nanjing, China). Finally, cDNA was amplified using an RT‒qPCR kit (Vazyme, Nanjing, China). RT‒qPCR of the target mRNAs was performed on a CFX96 real-time qPCR instrument (Bio-Rad, USA). The relative levels of different genes were represented as bar graphs by calculating the comparative 2^−△△Ct^. The primer sequences of the target genes are presented in Table 1.

**Table 1:** The primer sequences of the target genes

Gene name	Forward sequence (5'-3')	Reverse sequence (5'-3')
CCR1	TACTCTGGAAACACAGACTCACT	ACAGCAGTCTTTTGGCATGG
CCL3	CCAAGTCTTCTCAGCGCCA	CGGTTTCTCTTAGTCAGGAAAATGA
CCL4	CTGTGCAAACCTAACCCCGA	AGGGTCAGAGCCCATTGGT
CCL5	GTGCCCACGTCAAGGAGTAT	TCGAGTGACAAACACGACTG
CCL6	TCAAGCCGGGCATCATCTTT	CTGCCCTCCTTCTCAAGCAA
CCL7	CCCTGGGAAGCTGTTATCTTCAA	CTCGACCCACTTCTGATGGG
GAPDH	CCC​AGC​TTA​GGT​TCA​TCA​GG	ATC​TCC​ACT​TTG​CCA​CTG​C
MMP13	TGA​TGG​ACC​TTC​TGG​TCT​TCT​GG	CAT​CCA​CAT​GGT​TGG​GAA​GTT​CT
Collagen II	GGC​CAG​GAT​GCC​CGA​AAA​TTA	CGC​ACC​CTT​TTC​TCC​CTT​GT
INOS	CTCCTGCCTCATGCCATTG	AGCTCATCCAGAGTGAGCTG
COX2	GATAACCGAGTCGTTCTGCC	AATCCTGGTCGGTTTGATGC

### SA-β-gal staining

The senescence level of chondrocytes was evaluated with a senescence-associated β-galactosidase (SA-β-gal) staining kit (Beyotime, Shanghai, China). According to the manufacturer’s instructions, cells were stained with different concentrations of blue and observed under an optical microscope.

### Animal experiments

All 32 C57BL/6J mice included were male and were obtained from Gempharma Tech (Nanjing, China). The mice had no genetic modifications and had not undergone any prior experimental use. To establish a mouse osteoarthritis model, we performed medial meniscus destabilization surgery (Glasson et al. 2007). The detailed procedure involved shaving the hair around the knee joint, making an incision in the anterior medial skin of the knee joint using a surgical scalpel, opening the joint capsule, exposing the anterior foot of the medial meniscus, and cutting the medial meniscus anterior horn using a fine-tip modeling tool to confirm its destabilized state. The joint capsule and skin were sutured. The mice were randomly divided into four groups (n = 8 per group), the sham group, the DMM group and the DMM + BX471 (1.5 or 3 mg/kg) group, with the surgical site being the right knee joint. After inducing anesthesia with 1% pentobarbital sodium, the sham surgery group underwent no further procedures after the joint capsule was opened, and the incision was closed. The other three groups of mice underwent the aforementioned surgical procedures. Postsurgery, the mice were placed in a lateral position in empty cages and, upon complete recovery from anesthesia, were transferred to cages containing food and drinking water. We did not apply pain management or antibiotics. On the 7th day postsurgery, mice from different groups underwent intra-articular injections into the joint space. The BX471 concentrations used were 1.5 mg/kg and 3 mg/kg. Prior to injection, the mice were weighed to calculate the injection dosage. The sham surgery group and DMM group received injections of physiological saline. Subsequently, injections into the joint space were administered every 7 days for a total of five consecutive injections. Throughout this period, the mice were observed, and in the event of severe limping or noticeable weight loss due to impaired knee joint function affecting food intake, humane euthanasia was performed using CO2. All the right legs were separated for the next experiments. All the animals used in this study were approved by The Institutional Animal Care and Use Committee of Tongji Hospital (Wuhan, China).

### Microcomputed tomography (μ-CT)

We scanned all the right knee joints of the mice by using a μ-Computed Tomography system (Scanco Medical, Bassersdorf, Switzerland). We obtained 21.0 μm resolution μ-CT images at 70 kV and 113 μA and generated 3D images.

### Histological and immunohistochemistry assessment

The separated joints were soaked in 4% paraformaldehyde for 48 h and then decalcified with 10% EDTA for 20 days. After paraffin embedding, the samples were sliced into 5 μm sections and then stained with hematoxylin–eosin (H&E) and Safranin O/Fast Green. The severity of cartilage damage was assessed according to the Osteoarthritis Research Society International (OARSI) guidelines (Glasson et al. 2010). The sections were blocked with 5% BSA for 1 h after deparaffinization. The samples were incubated with primary antibodies against CCR1, collagen II and MMP13 at 4 °C overnight. After they were incubated with secondary antibodies, the samples were observed, and images were acquired with a microscope.

### Statistical analysis

GraphPad Prism v.8.4.0 software was used to analyze the experimental data. All experiments were performed independently at least three times, and the data are presented as the means ± standard deviations (SDs). All the data were analyzed using the Shapiro‒Wilk test for normally distributed data and Levene’s test for homogeneity of variance. The significant differences between normally distributed groups were analyzed by one-way analysis of variance (ANOVA). Nonparametric data (OARSI scores) were compared using the Kruskal‒Wallis H test. A *p* value < 0.05 was considered to indicate statistical significance.

## Results

### Elevated chemokine and CCR1 expression in IL-1β-induced mouse chondrocytes and DMM OA models

**Fig. 1:**
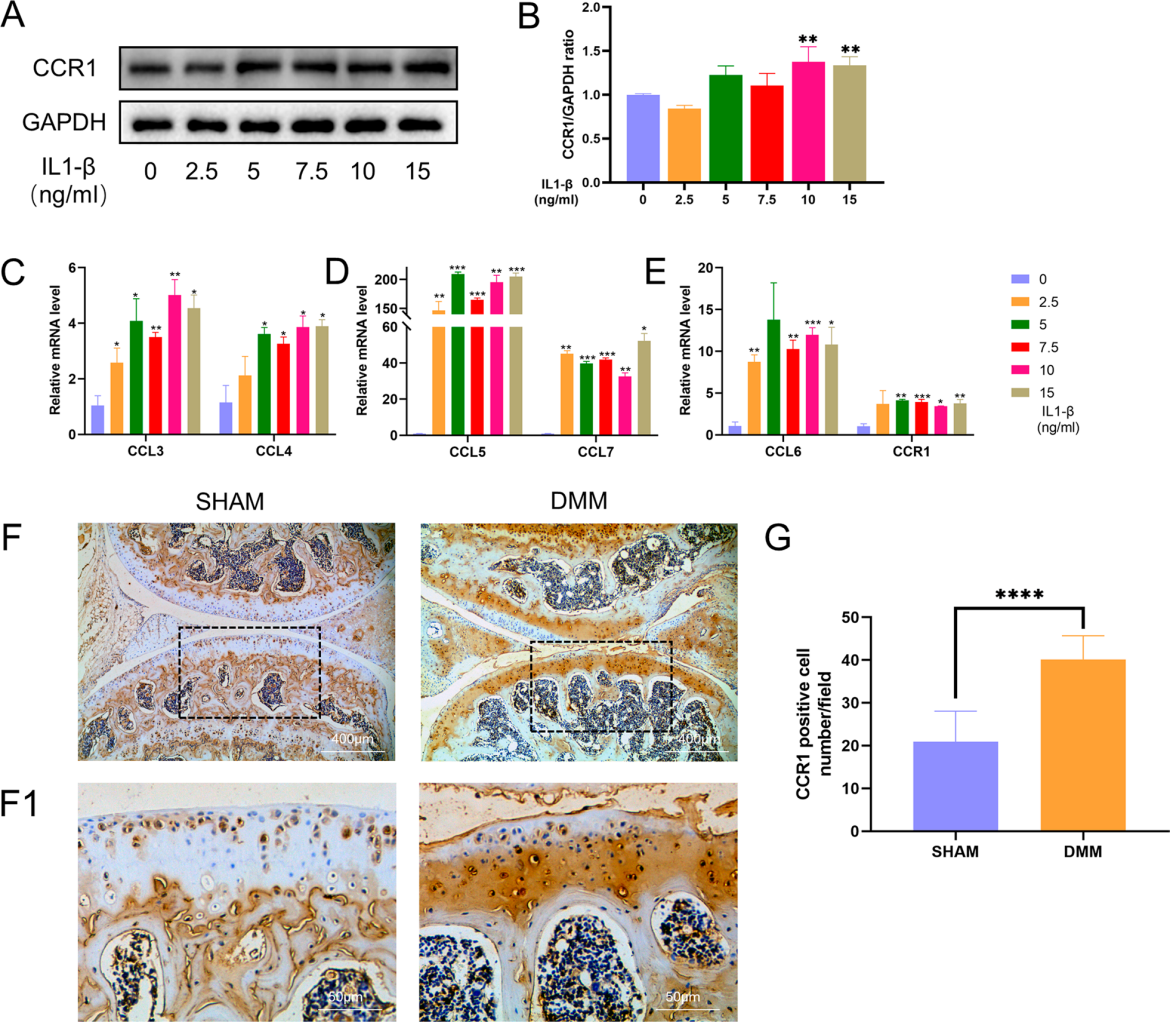
The expression of CCR1 in mouse chondrocytes. Chondrocytes were stimulated by IL-1β for 24 h. **A** Western blot was employed to determine the expression of CCR1. **B** Relative protein expression was qualified by ImageJ software, GAPDH was used as the internal control. **C**–**E** The mRNA expression profiles of CCR1 and CCL3-7 were quantified by semi-quantitative RT-PCR using GAPDH as an internal control. **F** Immunohistochemistry staining of CCR1 (scale bar 400 μm and 50 μm). **G** Number of CCR1 positive cells per field under 100-time magnification. The values are shown as means ± SD of triplicate experiments. GAPDH served as the housekeeping gene. *p < 0.05, **p < 0.01 and ***p < 0.001 compared with the negative control (NC) group. ****p < 0.0001 compared with the SHAM group

As shown in Fig. 1A, B, in IL-1β-stimulated mouse chondrocytes treated with 10 ng/ml and 15 ng/ml IL-1β, CCR1 expression increased by approximately 40% compared to the baseline level. As depicted in Fig. 1C, E, key ligands of CCR1, namely, CCL3, CCL4, CCL5, CCL6, and CCL7, exhibited upregulated expression under IL-1β stimulation. In particular, CCL5 and CCL7 were substantially increased, reaching several-fold higher expression levels than those in the control group. According to the results of the animal experiments (Fig. 1F, G), CCR1-positive chondrocytes were approximately twofold more abundant in the DMM group than in the sham surgery group.

### BX471 did not affect mouse chondrocyte viability

**Fig. 2:**
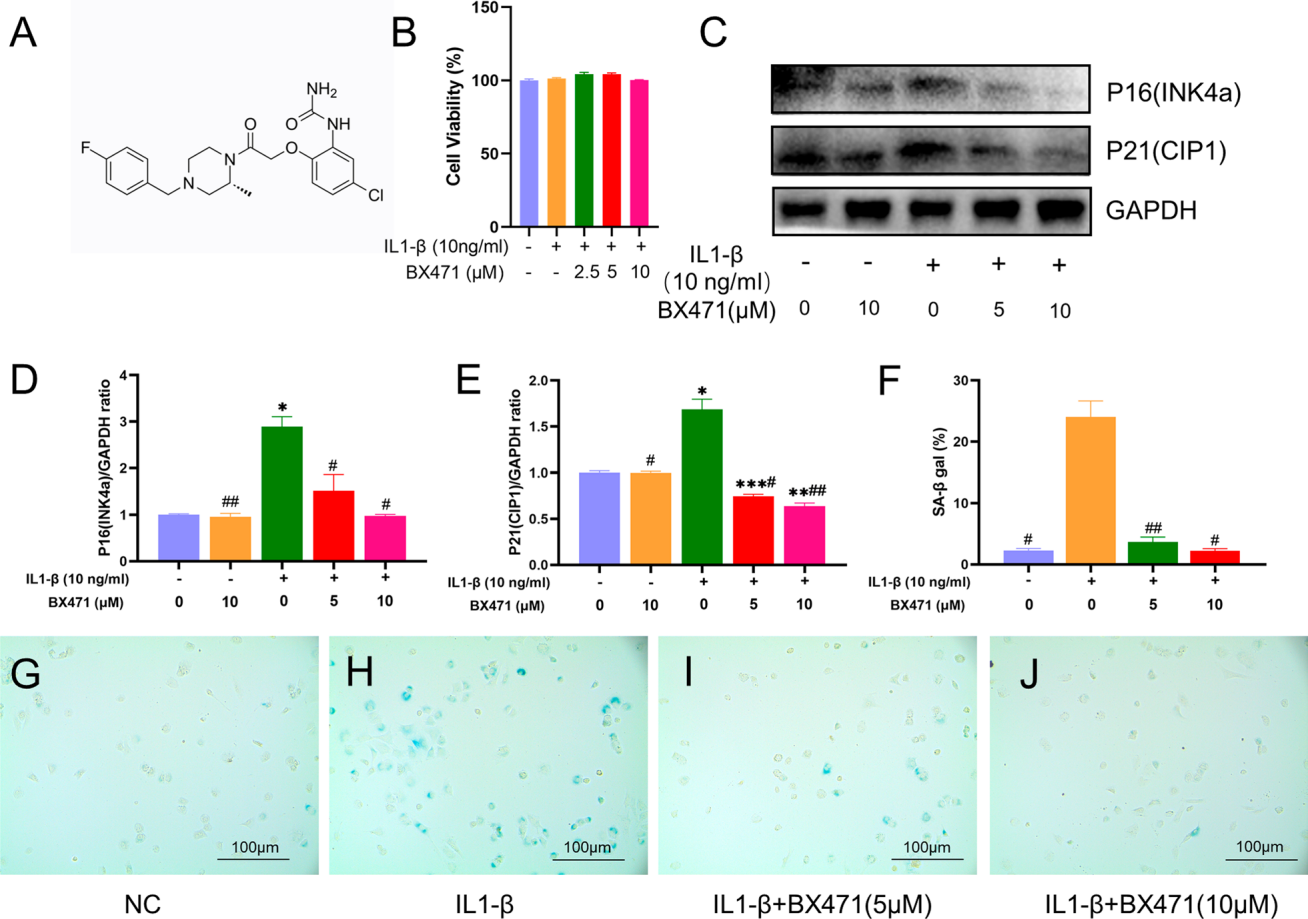
BX471 did not impact the viability of mouse chondrocytes and it could mitigate the IL1-induced chondrocyte senescence. **A** Chemical structure of BX471. **B** Cell viability was determined by CCK-8 assay. **C** Western blot was employed to determine the expression of P16INK4a and P21CIP1. **D**, **E** Relative protein expression was qualified by ImageJ software, GAPDH was used as the internal control. **F** The SA-β-gal activity was detected by SA-β-gal staining of senescent cells. **G**–**J** Quantitative analysis of SA-β-gal positive cell number (scale bar 400 μm). The values are shown as means ± SD of triplicate experiments. GAPDH served as the housekeeping gene. *p < 0.05, **p < 0.01 and ***p < 0.001 compared with the NC group; ^#^p < 0.05 and ^##^p < 0.01 compared with the IL-1β group

Stimulation with BX471 at concentrations of 2.5, 5, and 10 μM, with or without IL-1β (10 ng/ml) for 24 h, revealed, through CCK-8 kit assessment (Fig. 2B), that the various concentrations of BX471 did not influence cell viability. Subsequently, 5 and 10 μM BX471 were selected for subsequent experiments.

### CCR1 inhibition alleviated IL1-induced chondrocyte senescence

As shown in Fig. 2C–E, IL-1β-induced chondrocytes exhibited enhanced expression of the senescence markers P16INK4a and P21CIP1, indicating chondrocyte senescence. BX471 significantly attenuated the excessive expression of these markers. Senescence-associated β-galactosidase (SA-β-gal) activity, which was elevated in aging chondrocytes, returned to baseline levels upon BX471 treatment (Fig. 2F–H).

### CCR1 inhibition alleviated IL-1β-induced aggrecan and SOX9 degeneration in mouse chondrocytes

**Fig. 3:**
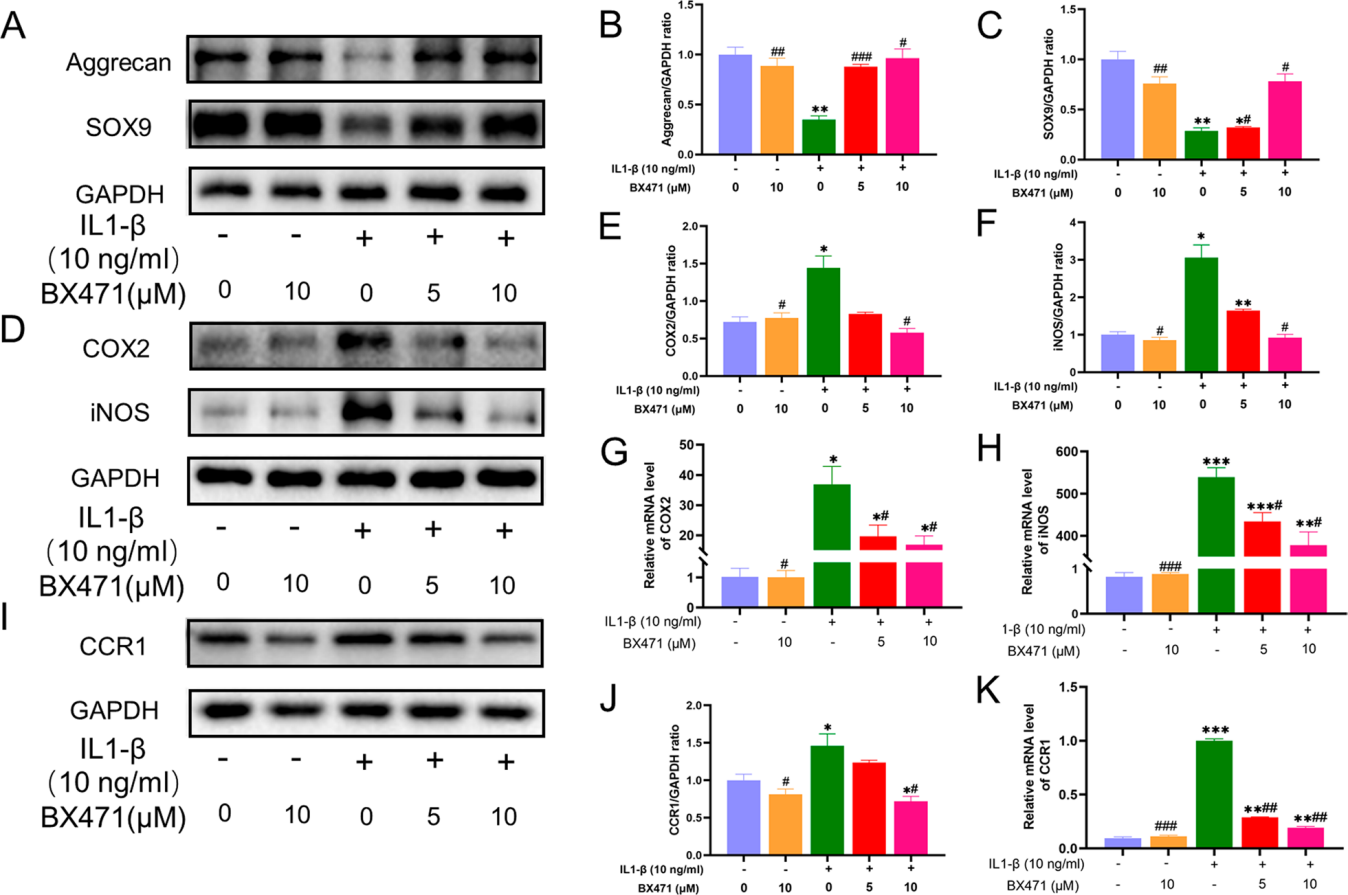
Inhibition of CCR1 alleviated the degeneration of Aggrecan and SOX9 and moderated inflammatory cytokines induced by IL-1β in mouse chondrocytes. BX471 down-regulated the expression of IL-1β-induced CRR1. **A** Western blot result of Aggrecan and SOX9. **B**, **C** Quantification analysis of western blot results, GAPDH was regarded as an internal control. **D** Western blot result of COX-2 and iNOS. **E**, **F** Quantification analysis of western blot results, GAPDH was regarded as an internal control. **G**, **H** The mRNA expression profiles of COX-2 and iNOS were quantified by semi-quantitative RT-PCR using GAPDH as an internal control. **I** Western blot result of CCR1. **J** Quantification analysis of western blot results, GAPDH was regarded as an internal control. **K** The mRNA expression profile of CCR1 was quantified by semi-quantitative RT-PCR using GAPDH as an internal control. GAPDH served as the housekeeping gene. The values are shown as means ± SD of triplicate experiments. *p < 0.05, **p < 0.01 and ***p < 0.001 compared with the NC group; ^#^p < 0.05, ^##^p < 0.01 and ^###^p < 0.001 compared with the IL-1β group

Aggrecan is a component of the extracellular matrix of chondrocytes and is an indicator of cell metabolism. The transcription factor SOX9 is a key factor in cartilage growth and development and can inhibit chondrocyte dedifferentiation to maintain the health of articular cartilage. IL-1β treatment for 24 h resulted in an approximately 70% decrease in the protein expression of AGG and SOX9 compared to baseline levels. However, inhibiting CCR1 restored AGG expression close to baseline levels and increased SOX9 expression to approximately 75% of baseline levels (Fig. 3A–C).

### CCR1 inhibition moderated IL-1β-induced inflammatory indicators in mouse chondrocytes

IL-1β induction significantly increased the mRNA levels of COX2 and iNOS by several-fold, which were reduced to approximately 50% of their peak levels with BX471 treatment. At the protein level, IL-1β-induced COX2 and iNOS expression nearly tripled, but BX471 reduced their expression to baseline levels (Fig. 3D–H).

### BX471 downregulates IL-1β-induced ccr1 expression in mouse chondrocytes

Treatment with 10 ng/ml IL-1β upregulated CCR1 expression in mouse chondrocytes, and this effect was reversed by BX471 at both the transcriptional and translational levels (Fig. 3I–K).

### CCR1 inhibition mitigated IL-1β-induced collagen II degeneration in mouse chondrocytes

**Fig. 4:**
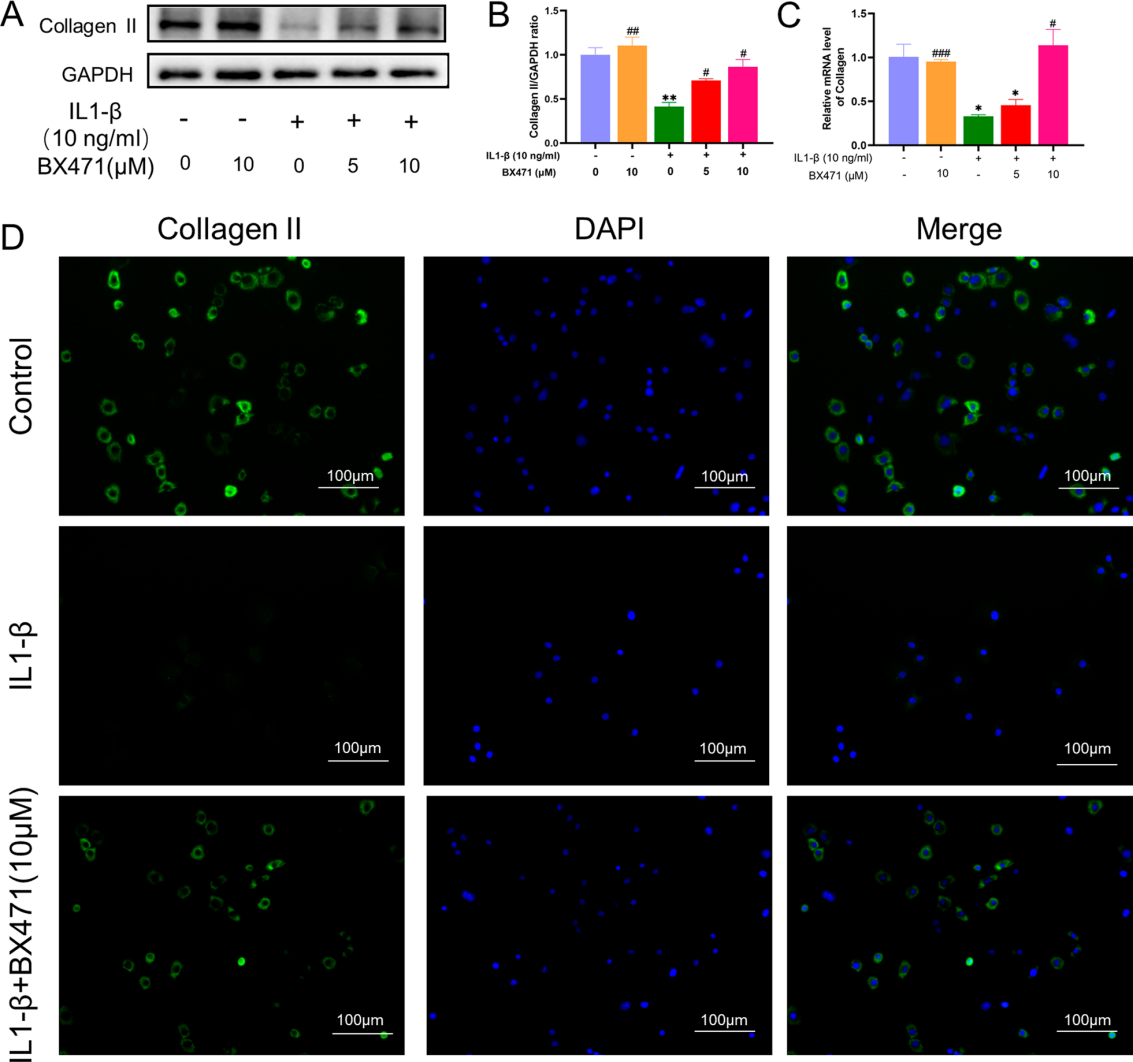
Inhibition of CCR1 mitigated the degeneration of Collagen II induced by IL-1β in mouse chondrocytes. **A** Western blotting result of Collagen II. **B** Expression of Collagen II was qualified by ImageJ software, GAPDH was regarded as an internal control. **C** The mRNA expression profile was quantified by semi-quantitative RT-PCR using GAPDH as an internal control. **D** Collagen II expression was determined by immunofluorescence staining (scale bar 100 μm). GAPDH served as the housekeeping gene. The values are shown as means ± SD of triplicate experiments. *p < 0.05 and **p < 0.01 compared with the NC group; ^#^p < 0.05,^##^p < 0.01 and ^###^p < 0.001 compared with the IL-1β group

IL-1β-treated chondrocytes exhibited significant degradation of collagen II, the main marker of chondrocytes. CCR1 inhibition with BX471 restored the collagen II concentration to approximately 90% of the baseline level, as shown in Fig. 4. The fluorescence results further indicated that compared with that in the IL-1β-treated group, collagen II (green light) in the BX471-treated group was more intense.

### CCR1 inhibition suppressed the IL-1β-induced MMP13 elevation in mouse chondrocytes

**Fig. 5:**
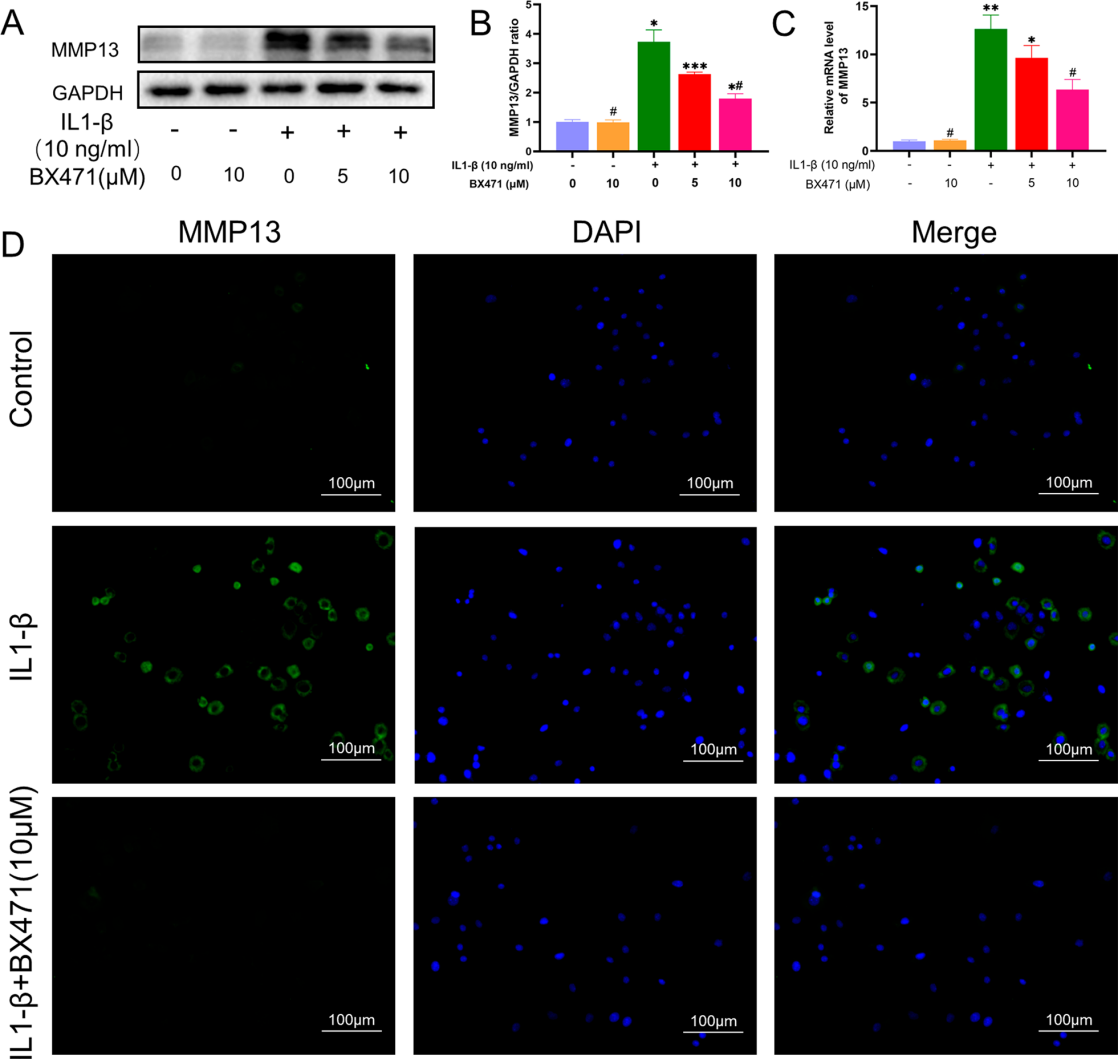
Inhibition of CCR1 mitigated the degeneration of MMP13 induced by IL-1β in mouse chondrocytes. **A** Western blotting result of MMP13. **B** Expression of MMP13 was qualified by ImageJ software, GAPDH was regarded as an internal control. **C** The mRNA expression profiles of MMP13 was quantified by semi-quantitative RT-PCR using GAPDH as an internal control. **D** MMP13 expression was determined by immunofluorescence staining (scale bar 100 μm). GAPDH served as the housekeeping gene. The values are shown as means ± SD of triplicate experiments. *p < 0.05, **p < 0.01 and ***p < 0.001 compared with the NC group; ^#^p < 0.05 compared with the IL-1β group

IL-1β treatment for 24 h significantly increased the expression of MMP13, a representative marker of MMPs. BX471 reduced MMP13 expression by approximately 50% at both the transcriptional and translational levels, as depicted in Fig. 5. The fluorescence results were consistent with this effect.

### CCR1 inhibition suppressed IL-1β-induced activation of the MAPK pathway in mouse chondrocytes

**Fig. 6:**
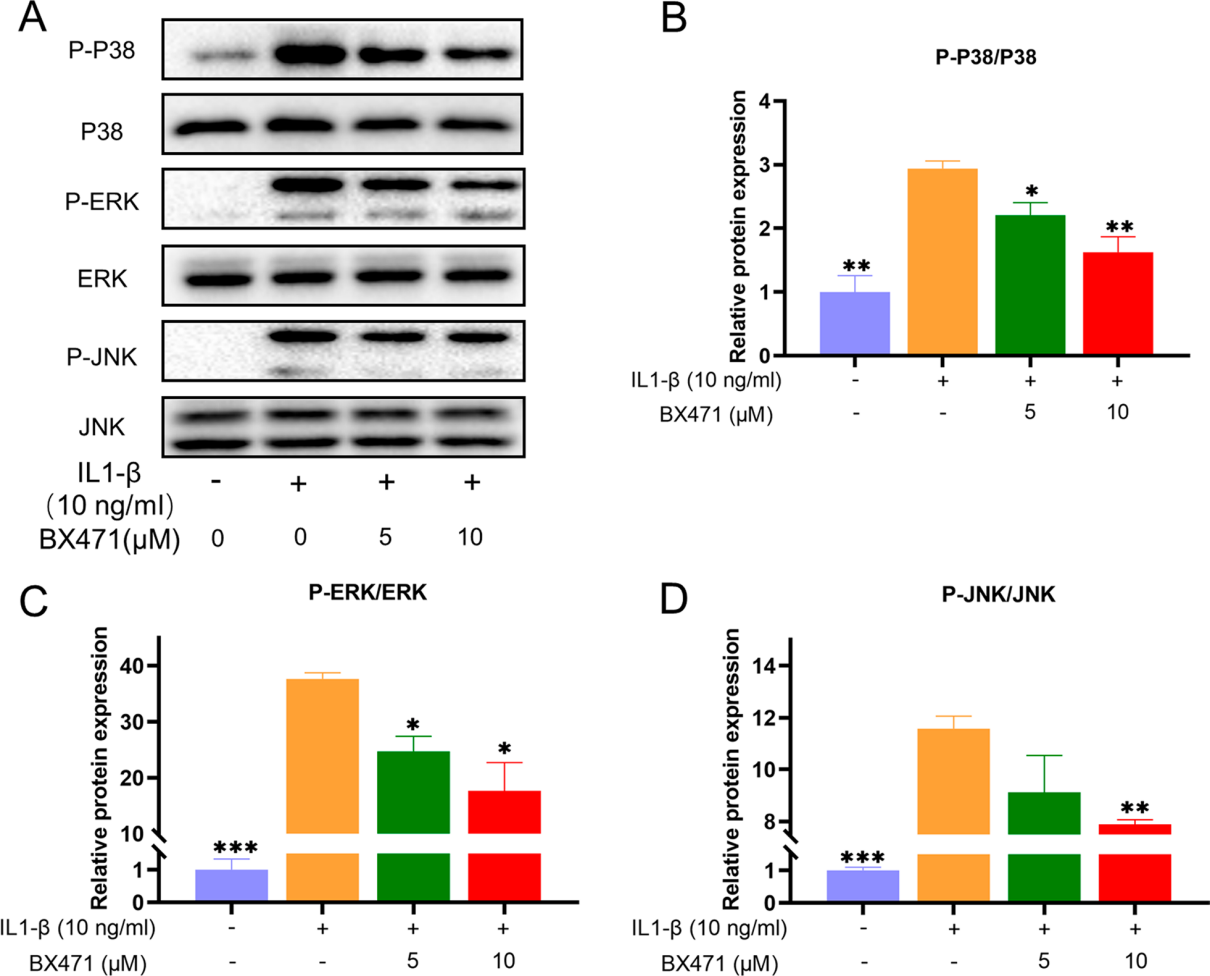
Inhibition of CCR1 suppressed IL-1β-induced activation of MAPK pathway in mouse chondrocytes. **A** Cells were exposed to BX471 (5 or 10 μM) for 24 h with or without IL-1β (10 ng/ml) for 30 min. Protein levels of P-P38, P38, P-ERK, ERK, P-JNK and JNK were detected by Western blot. **B** Relative protein expression was qualified by ImageJ software, P38 was used as the loading control. **C** Relative protein expression was qualified by ImageJ software, ERK was used as the loading control. **D** Relative protein expression was qualified by ImageJ software, JNK was used as the loading control. The values are shown as means ± SD of triplicate experiments. *p < 0.05, **p < 0.01 and ***p < 0.001 vs. IL-1β group

The IL-1β-induced phosphorylation of P38, ERK, and JNK in the MAPK pathway, which is crucial in OA, was mitigated by approximately 50% with BX471 treatment, as illustrated in Fig. 6.

### CCR1 inhibition increased PPAR-γ expression, and GW9662 weakened the OA remission effect of BX471

**Fig. 7:**
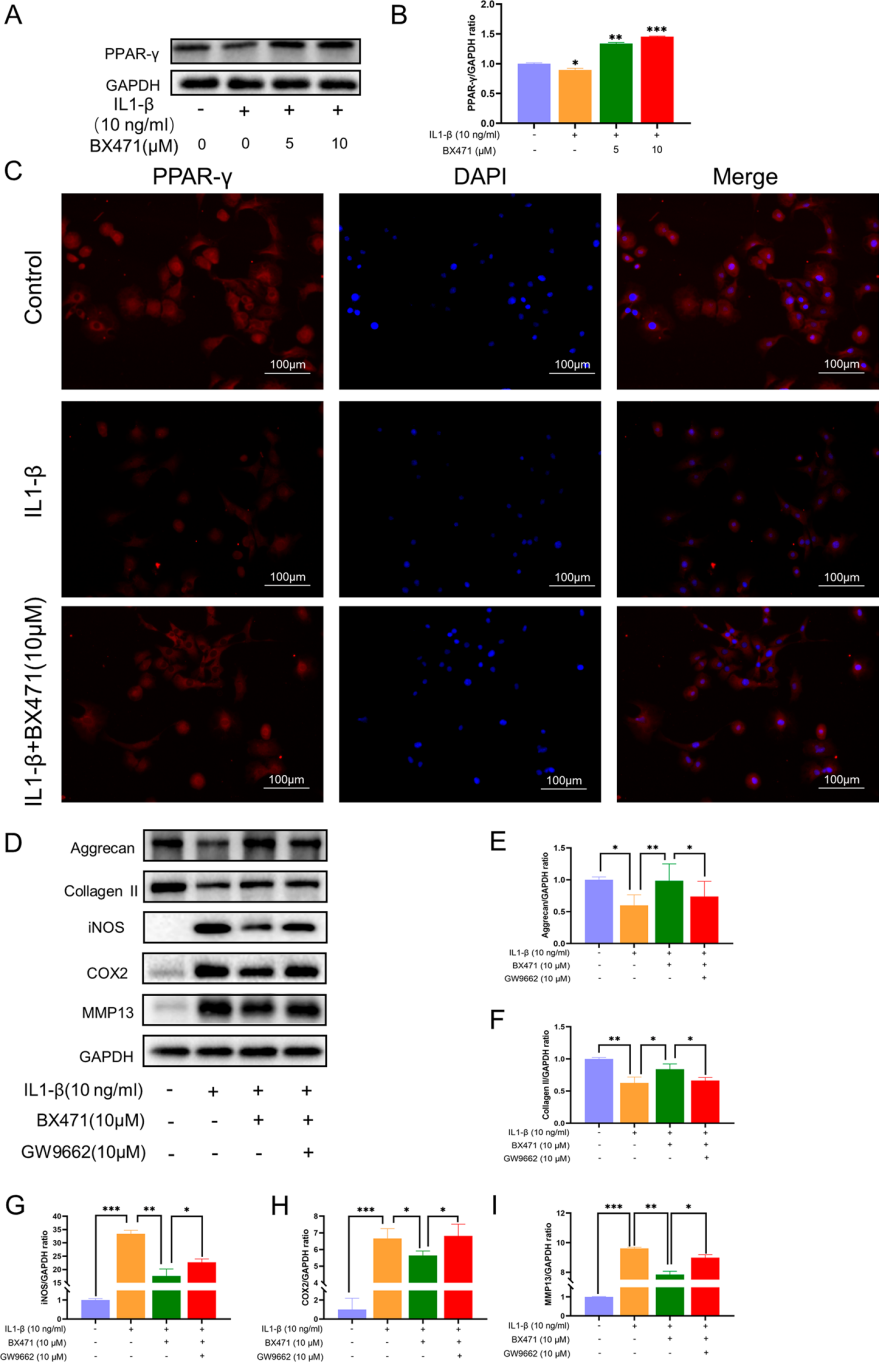
Inhibition of CCR1 increased the expression of PPAR-γ and GW9662 weakened the OA remission effect of BX471. **A** Western blotting result of PPAR-γ. **B** Expression of PPAR-γ was qualified by ImageJ software, GAPDH was regarded as an internal control. **C** PPAR-γ expression was determined by immunofluorescence staining (scale bar 100 μm). *p < 0.05, and ***p < 0.001 vs. IL-1β group. **D** Pretreated with GW9662 (10 μM) for 2 h, cells were treated to IL-1β (10 ng/ml) and/or BX471 (10 μM) for 24 h. Protein levels of Collagen II, Aggrecan, MMP13, iNOS and COX-2 were detected by Western blot. **E**–**I** Relative protein expression was qualified by ImageJ software. GAPDH served as the housekeeping gene. The values are shown as means ± SD of triplicate experiments; *p < 0.05, **p < 0.01 and ***p < 0.001

IL-1β treatment significantly decreased the expression of PPAR-γ (Fig. 7A, B), a known anti-OA factor (Vallée and Lecarpentier 2018; Montaigne et al. 2021), which was reversed by CCR1 inhibition. BX471 treatment resulted in increased PPAR-γ (red light) compared to that in the IL-1β-treated group (Fig. 7C). Validation experiments with GW9662 revealed decreased expression of aggrecan and collagen II and increased expression of COX-2, iNOS, and MMP13, suggesting that PPAR-γ plays a role in the regulation of OA-related phenotypes by BX471 (Fig. 7D–I).

### CCR1 inhibition ameliorated cartilage destruction in mouse DMM OA models

**Fig. 8:**
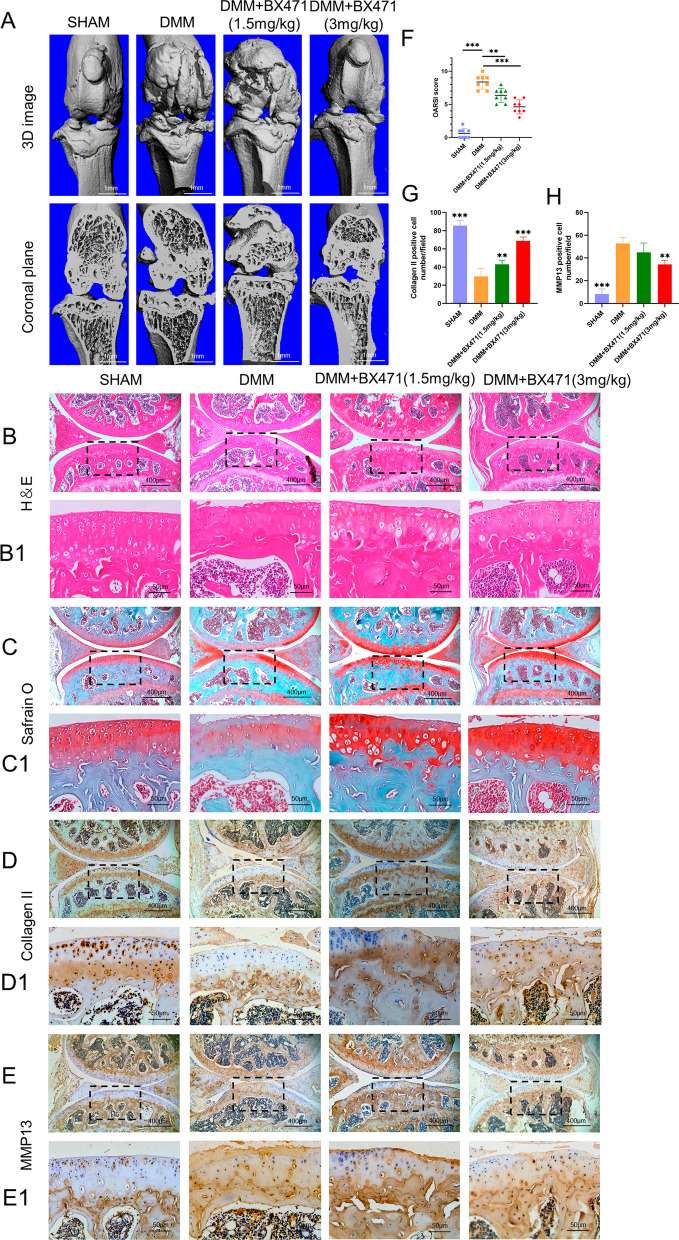
Inhibition of CCR1 attenuated the vitiation of cartilage on the mouse OA mode. **A** Representative images showed the 3D image and coronal views of the right knee and from SHAM, DMM and DMM + BX471 groups (scale bar 1 mm). **B** H&E staining of different groups. **C** safranin O/fast green staining of different groups. **D** Immunohistochemistry staining of Collagen II. **E** Immunohistochemistry staining of MMP13. Scale bar 400 μm and 50 μm. **F** The OARSI scores of each group. **p < 0.01 and ***p < 0.001 vs. DMM group. **G** Number of Collagen II positive cells per field under 100-time magnification. **H** Number of MMP13 positive cells per field under 100-time magnification. **p < 0.01 and ***p < 0.001 vs. DMM group

Macroscopic and microscopic examinations demonstrated that intra-articular injection of BX471 delayed OA development, as evidenced by reduced OA-related features and improved cartilage histology compared to those of the DMM group (Fig. 8). The number of MMP13-positive cells increased in the DMM group but decreased in the DMM + BX471 group, while the number of collagen II-positive cells exhibited the opposite trend.

## Discussion

Osteoarthritis is a degenerative condition that causes chronic joint pain, swelling, deformity, and restricted movement. With its prevalence escalating globally due to the aging population, finding safe and effective treatments for OA has become increasingly urgent (Bortoluzzi et al. 2018). Currently, nonsurgical OA management relies primarily on drug therapy, such as nonsteroidal anti-inflammatory drugs (NSAIDs) (Glyn-Jones et al. 2015; Abramoff and Caldera 2020), which may cause side effects such as gastrointestinal upset and ulceration. Understanding the pathogenesis of OA is essential, and studies underscore the close association of inflammation and cellular senescence with OA progression (Zheng et al. 2021; Kapoor et al. 2011; Rahmati et al. 2017; Rim et al. 2020).

Chemokines and their receptor families, which are integral players in various inflammatory metabolic diseases, have long been regarded as potential targets for immune disease treatment, particularly in the context of conditions such as rheumatoid arthritis, where immune system overactivation can be regulated (Lu et al. 2015; Dairaghi et al. 2011). Although CCR1 has been recognized as a crucial target in rheumatoid arthritis (RA) treatment, its role in osteoarthritis remains inadequately explored. Previous studies have reported inconsistent expression levels of CCR1 in the cartilage tissues of both healthy individuals and OA patients (Hopwood et al. 2009; Manferdini et al. 2020; Szekanecz and Koch 2016). However, the diverse outcomes across these studies have precluded a definitive conclusion. In our investigation, cellular experiments revealed the expression of CCR1 in mouse chondrocytes, and its upregulation was stimulated by the inflammatory cytokine IL-1β. RT‒qPCR results demonstrated that IL-1β induces chondrocytes to upregulate the expression of CCR1 and its major ligands (Haas et al. 2005). Moreover, the CCR1 inhibitor BX471 reduced the inflammatory response induced by IL-1β. These findings suggest that chondrocytes can secrete chemokines and express chemokine receptors, a property closely associated with IL-1β, the primary inflammatory cytokine in OA. Consequently, CCR1 has emerged as a potential target for OA treatment. To further elucidate the role of CCR1 in OA, we treated mouse chondrocytes with varying concentrations of IL-1β and BX471 and observed an increase in CCR1 expression with increasing IL-1β concentrations and a decrease with increasing BX471 concentrations.

IL-1β, a pivotal inflammatory factor in the onset and progression of OA, induces the progressive destruction of articular cartilage (Pelletier et al. 2001; Elsaid et al. 2015). IL-1β significantly upregulates the expression of zinc-dependent matrix metalloproteinases (MMPs) in chondrocytes, promoting the degradation of aggrecan and collagen II within the chondrocyte extracellular matrix (Kapoor et al. 2011). Concurrently, IL-1β also significantly upregulated the expression of the inflammatory factors iNOS and COX-2. The elevation of these factors induces changes in the intracellular inflammatory microenvironment (Glyn-Jones et al. 2015; Pelletier et al. 2001), fostering a self-perpetuating cycle of IL-1β secretion by the cells. Our study revealed the activation of CCR1 in chondrocytes, with 10 μM BX471 inhibiting CCR1 expression. Moreover, BX471 suppressed the IL-1β-induced expression of inflammatory metabolism-related proteins, including MMP13, iNOS, and COX-2. Additionally, BX471 inhibited the IL-1β-induced degradation of the extracellular matrix proteins Aggrecan and Collagen II. Consequently, we hypothesized that inhibiting CCR1 could safeguard articular cartilage by inhibiting the inflammatory metabolic response of chondrocytes.

Chondrocyte aging is intricately linked to OA development (Rahmati et al. 2017). Studies have indicated that chondrocyte senescence-associated β-galactosidase (SA-β-gal) activity increases with age (Martin and Buckwalter 2001). Gao's research confirmed the correlation between SA-β-gal activity and OA severity (Gao et al. 2016). Additionally, Xu reported that injecting senescent cells into the knee joint of mice resulted in severe articular cartilage damage (Xu et al. 2017). In our study, the use of BX471 inhibited the expression of the aging marker genes P16INK4a and P21CIP1 and the activity of SA-β-gal. Therefore, we propose that inhibiting CCR1 could protect cartilage by mitigating chondrocyte senescence.

The MAPK signaling pathway plays a crucial role in the OA process (Zhou et al. 2019; Sondergaard et al. 2010). The P38/MAPK signaling pathway is implicated in chondrocyte senescence, and its activation upregulates the expression of inflammatory proteins (COX-2 and iNOS) while downregulating the expression of synthetic proteins (AGG and Collagen II), thereby promoting cartilage destruction and joint inflammatory responses. Our study demonstrated that IL-1β-induced MAPK signaling pathway activation could be inhibited by the CCR1 inhibitor BX471, revealing the protective mechanism of CCR1 inhibition in preserving articular cartilage.

**Fig. 9:**
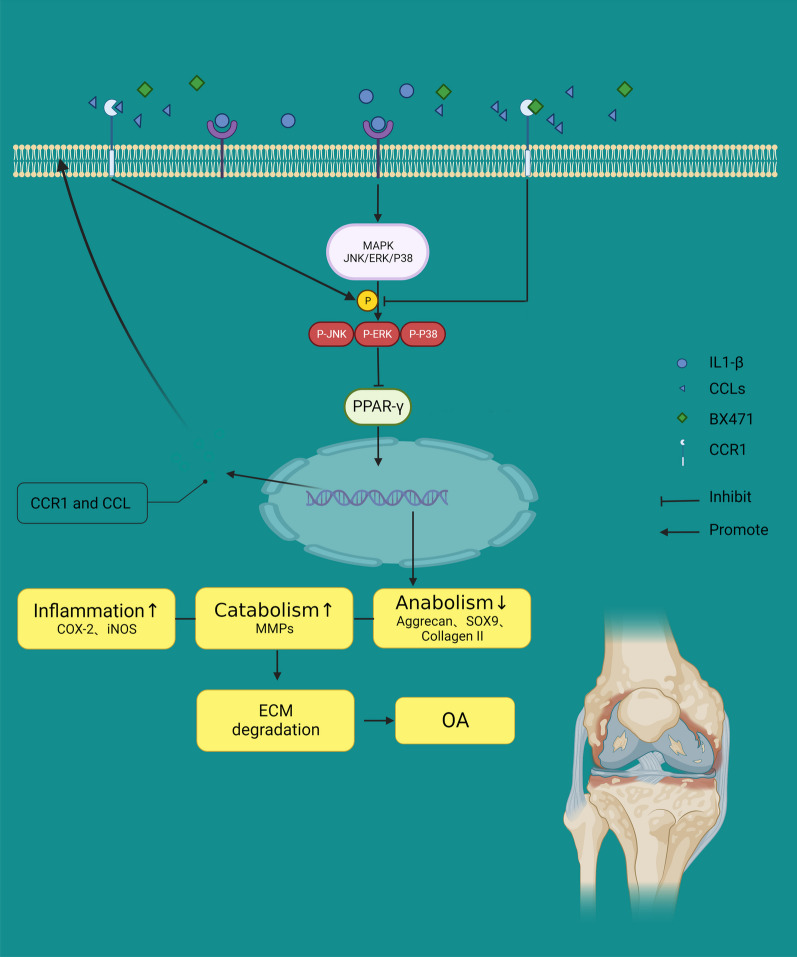
The mechanism of Inhibition of CCR1 in primary articular chondrocytes and the DMM model. As can be seen from the scheme, inhibition of CCR1 by BX471 can suppress IL-1β induced expression of iNOS, COX-2 and MMP13, and degradation of Aggrecan and Collagen II by inhibiting MAPK signaling pathway and activating PPAR-γ

PPAR-γ has emerged as a critical therapeutic target for OA and is involved in regulating inflammatory responses, cell proliferation, and apoptosis (Vallée and Lecarpentier 2018; Montaigne et al. 2021). IL-1β induction downregulated PPAR-γ expression in chondrocytes, and its significant downregulation in OA chondrocytes contributed to oxidative stress injury (Abshirini et al. 2021; Chen et al. 2022). Elevated PPAR-γ expression alleviated oxidative stress damage and inhibited the expression of various inflammatory metabolic factors (COX-2 and iNOS), playing a protective role in articular cartilage in OA (Sheng et al. 2023). Our data indicated that the CCR1 inhibitor BX471 significantly upregulated the IL-1β-induced decrease in PPAR-γ expression. To further validate the role of PPAR-γ in delaying OA progression through CCR1 inhibition, we employed a selective PPAR-γ inhibitor, GW9662. The results revealed that inhibiting PPAR-γ partially weakened the protective effects of CCR1 inhibition, as evidenced by anabolic increases, catabolic decreases, and downregulated expression of inflammatory factors in chondrocytes. Therefore, we hypothesized that PPAR-γ is involved in OA protection through CCR1 inhibition (Fig. 9).

Limitations included the inability to conduct human experiments, making it crucial to validate these findings in adult human joint chondrocytes. The surgically induced models of OA cannot completely recapitulate natural disease, and the limitations of this study should be acknowledged. Future studies could benefit from the use of knockout mice and the inclusion of cartilage samples from normal individuals and OA patients. Another major limitation was that not all in vitro results could be translated to in vivo conditions.

## Conclusion

Our data clearly demonstrated the expression of CCR1 in chondrocytes and its upregulation under inflammatory stimuli in vitro and in a low-grade inflammatory microenvironment, such as osteoarthritis (OA). The small molecule CCR1 inhibitor BX471 effectively inhibited inflammatory responses and senescence while promoting synthetic metabolism and inhibiting catabolic metabolism in chondrocytes in vitro. Additionally, this protective effect on chondrocytes was achieved through the modulation of the MAPK signaling pathway and PPAR-γ. In vivo, BX471 protected cartilage and reduced osteophyte formation, suggesting the therapeutic potential of CCR1 inhibition in experimental osteoarthritis.

### Supplementary Information

Supplementary Material 1.

## Data Availability

The raw data supporting the conclusion of this article will be made available by the authors, without undue reservation.

## Abbreviations

AGG: Aggrecan
BSA: Albumin from bovine serum
CCR1: CC chemokine receptor 1
COX-2: Cyclooxygenases-2
H&E: Hematoxylin–eosin
IHC: Immunohistochemistry
iNOS: Inducible nitric oxide synthase
MMPS: Matrix metalloproteinases
μ-CT: Micro-computed Tomography
DMM: Medial meniscus destabilized
NC: Negative control
OA: Osteoarthritis
PPAR-γ: Peroxisome proliferator activated receptor-γ
PBS: Phosphate buffered saline
RA: Rheumatoid arthritis
SA-β-gal: Senescence associated β-galactosidase
SDS-PAGE: Sodium dodecyl sulfate–polyacrylamide gel electrophoresis
SD: Standard deviation
MMTL: The medial meniscus tibial collateral ligament
TBST: Tris Buffered Saline Tween
