# Supplementary material for: Inhibition of CC chemokine receptor 1 ameliorates osteoarthritis in mouse by activating PPAR-γ
Source: Mol Med. 2024 Jun 3;30:74. doi: 10.1186/s10020-024-00823-w (PMC11149222; doi:10.1186/s10020-024-00823-w)
Supplement: Supplementary file 1 — Supplementary Material 1. [file 10020_2024_823_MOESM1_ESM.pptx]

## Slide 1
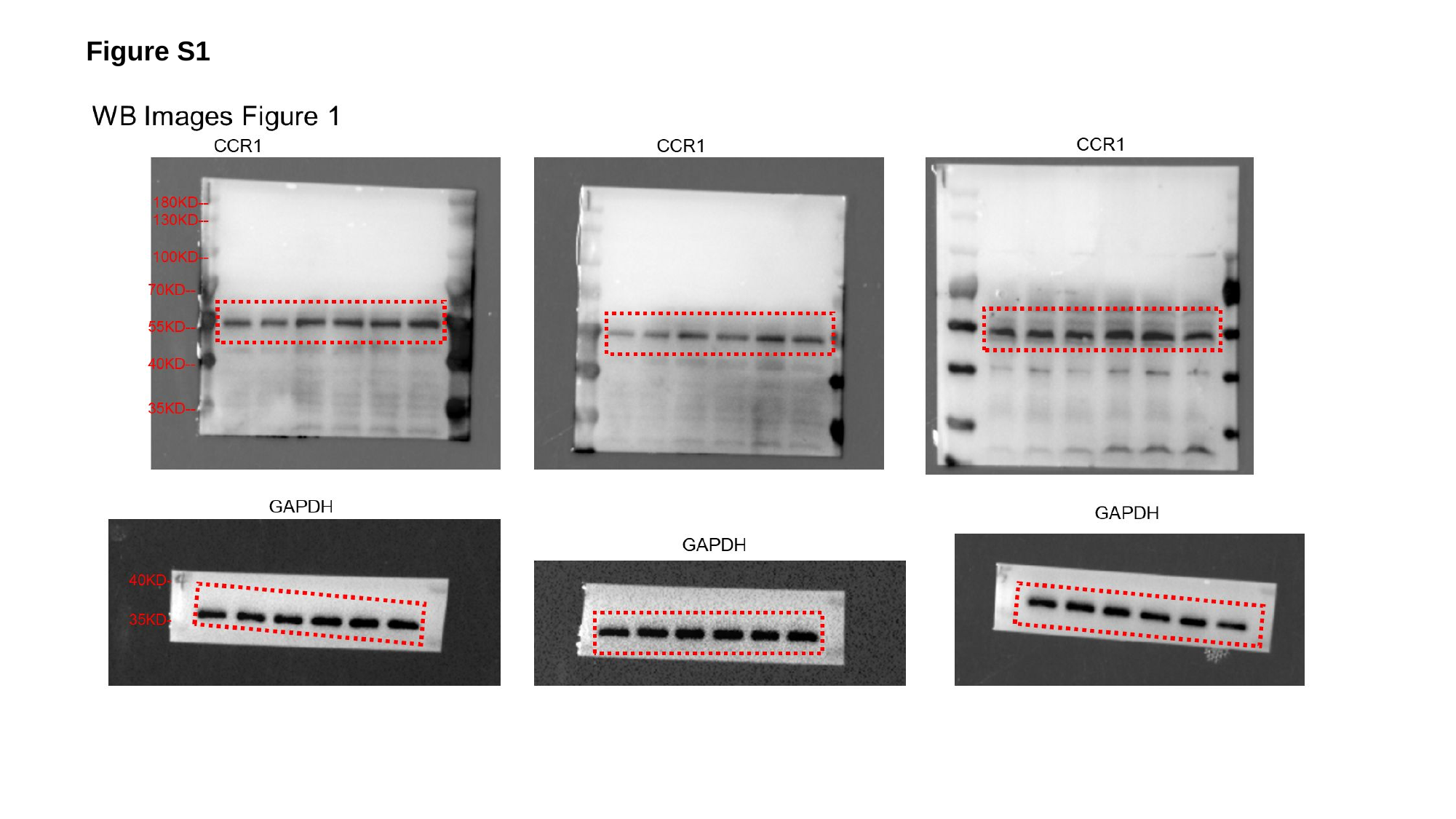

Figure S1

## Slide 2
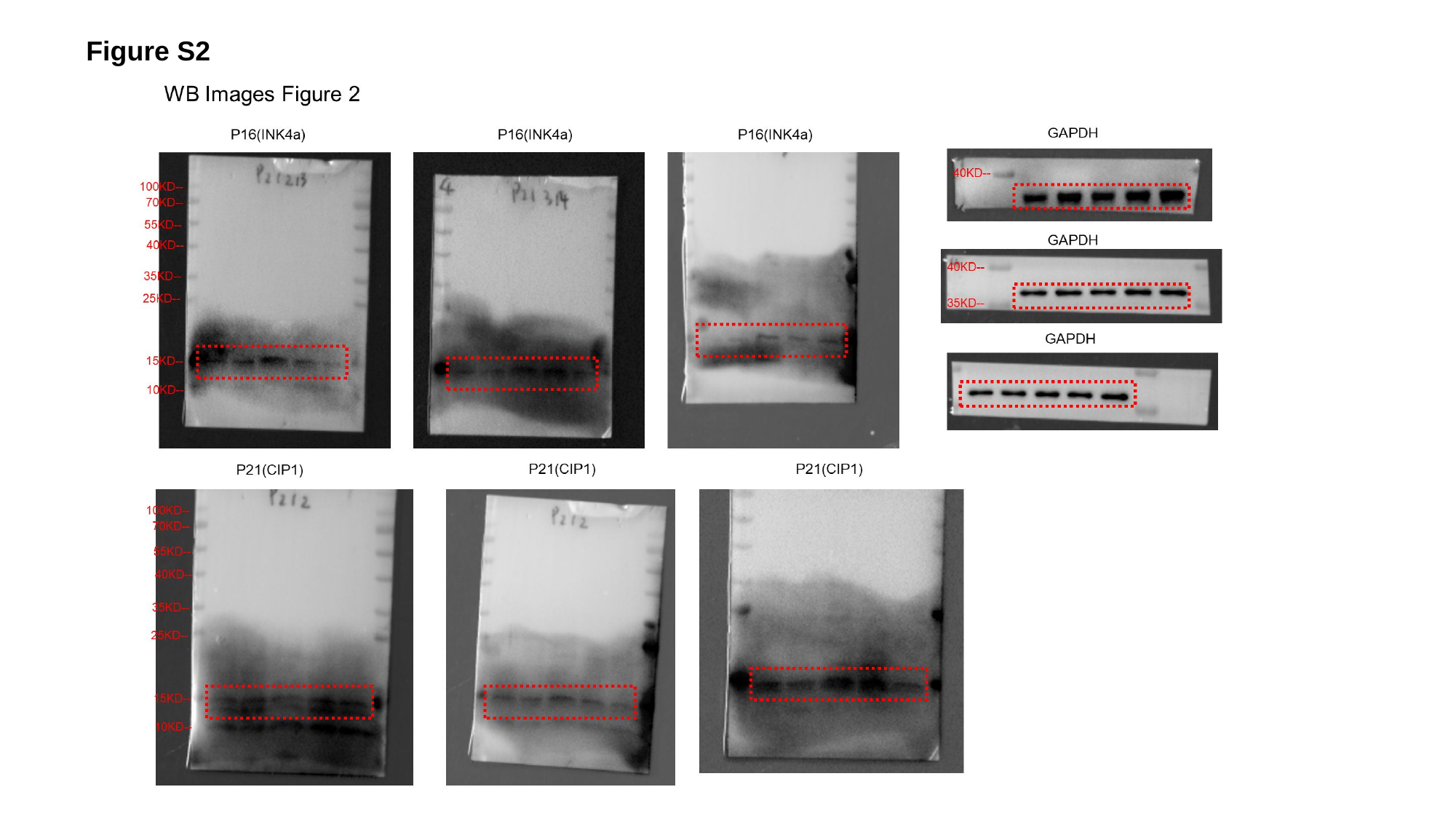

Figure S2

## Slide 3
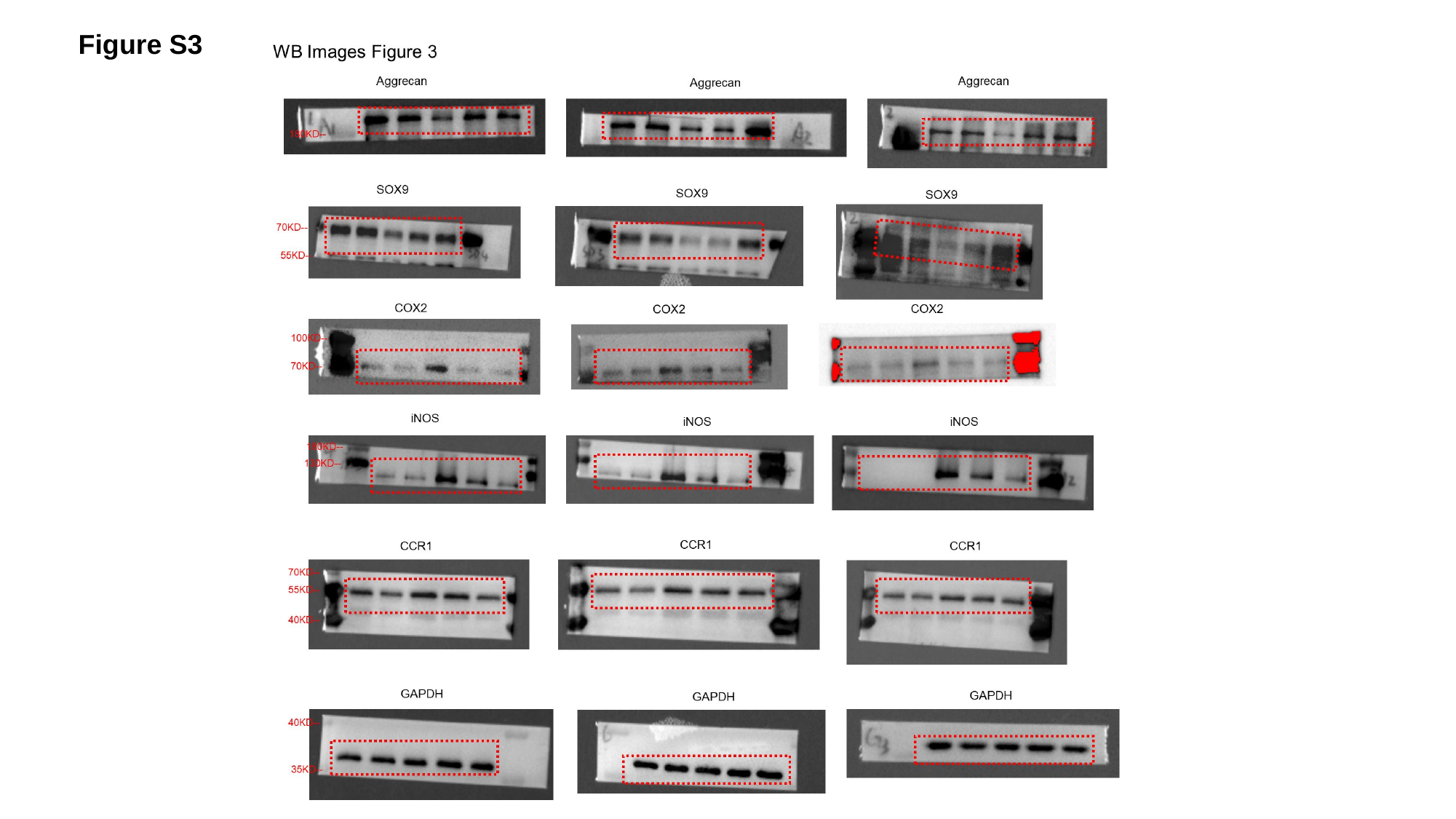

Figure S3

## Slide 4
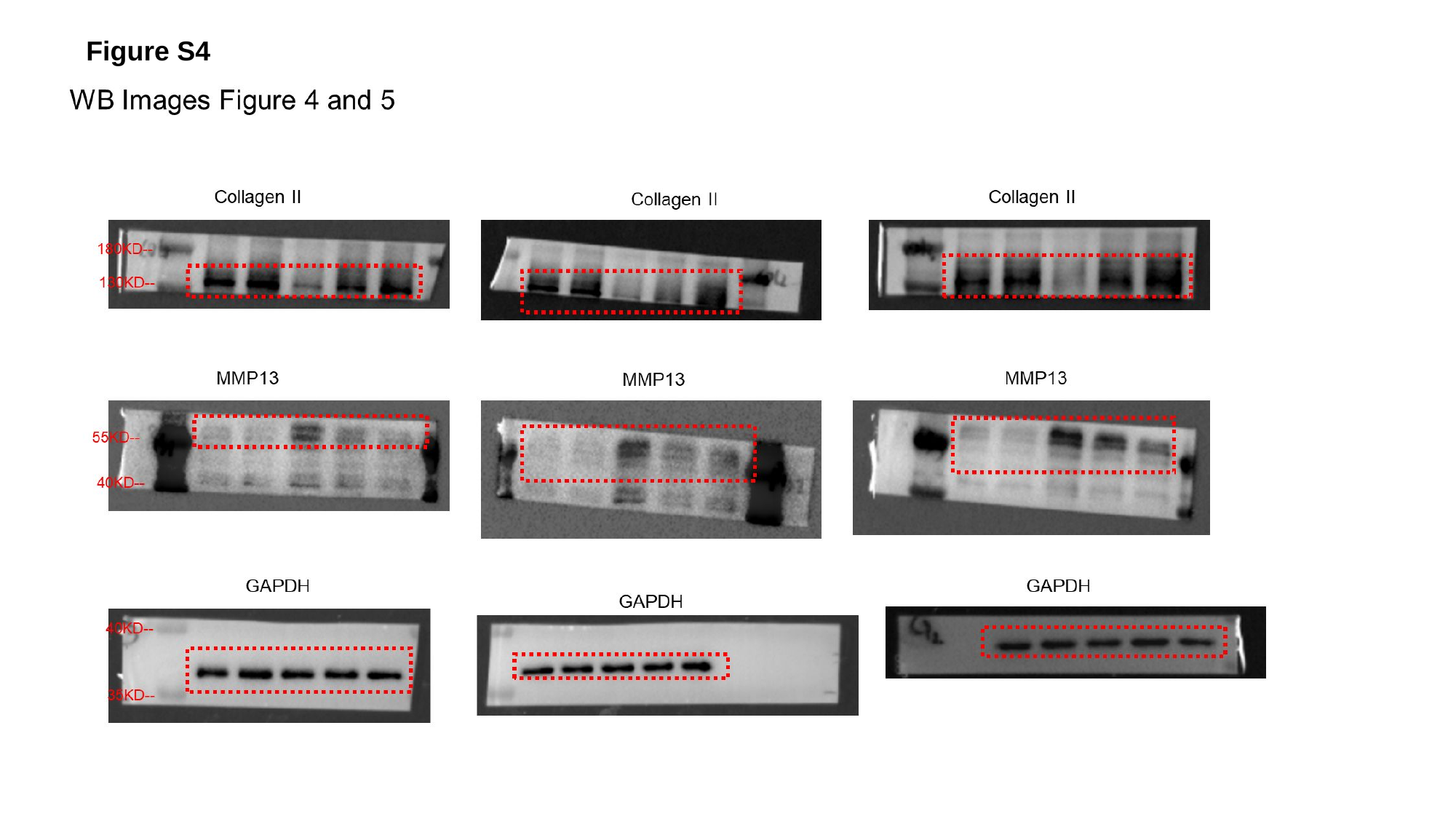

Figure S4

## Slide 5
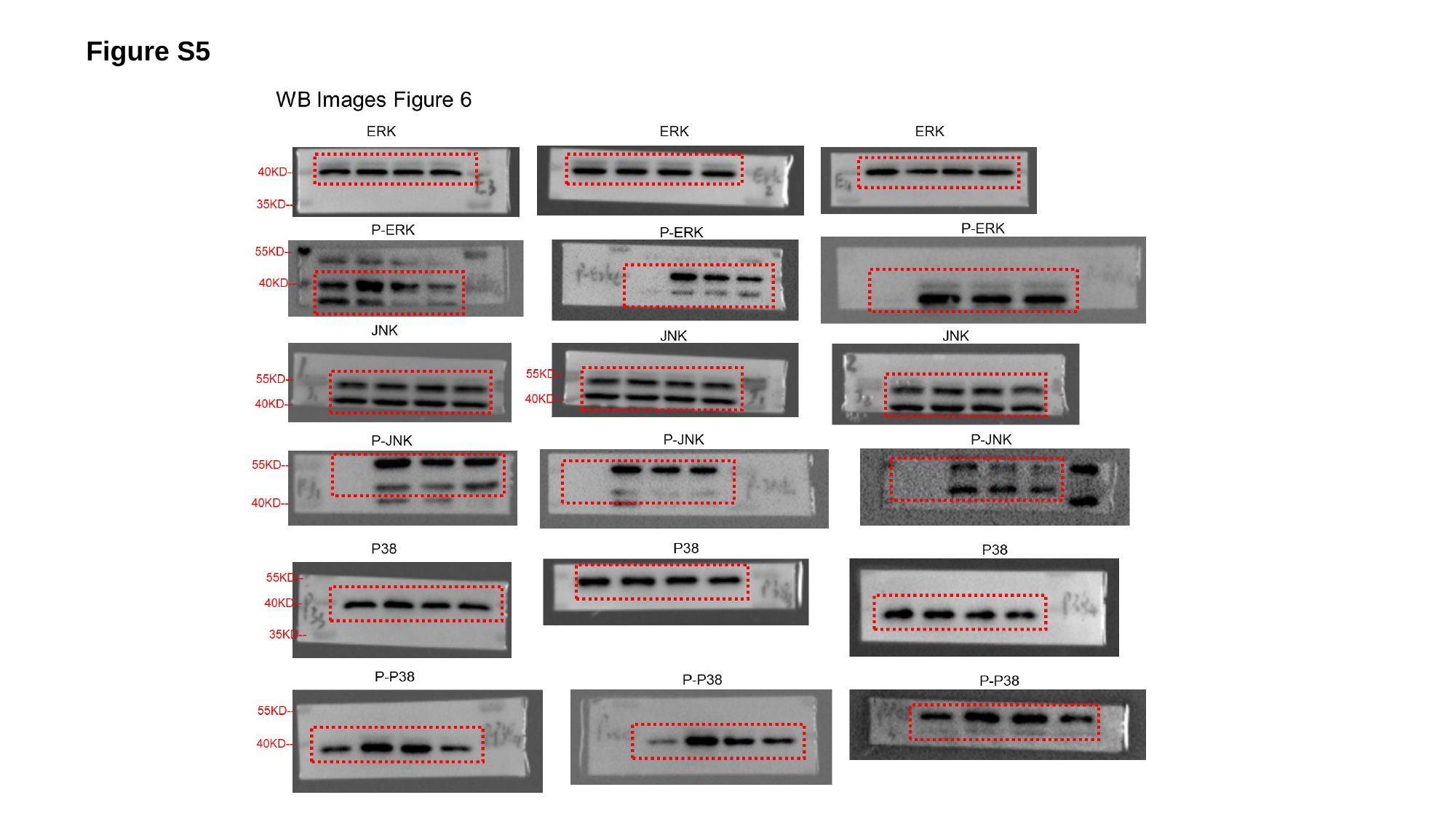

Figure S5

## Slide 6
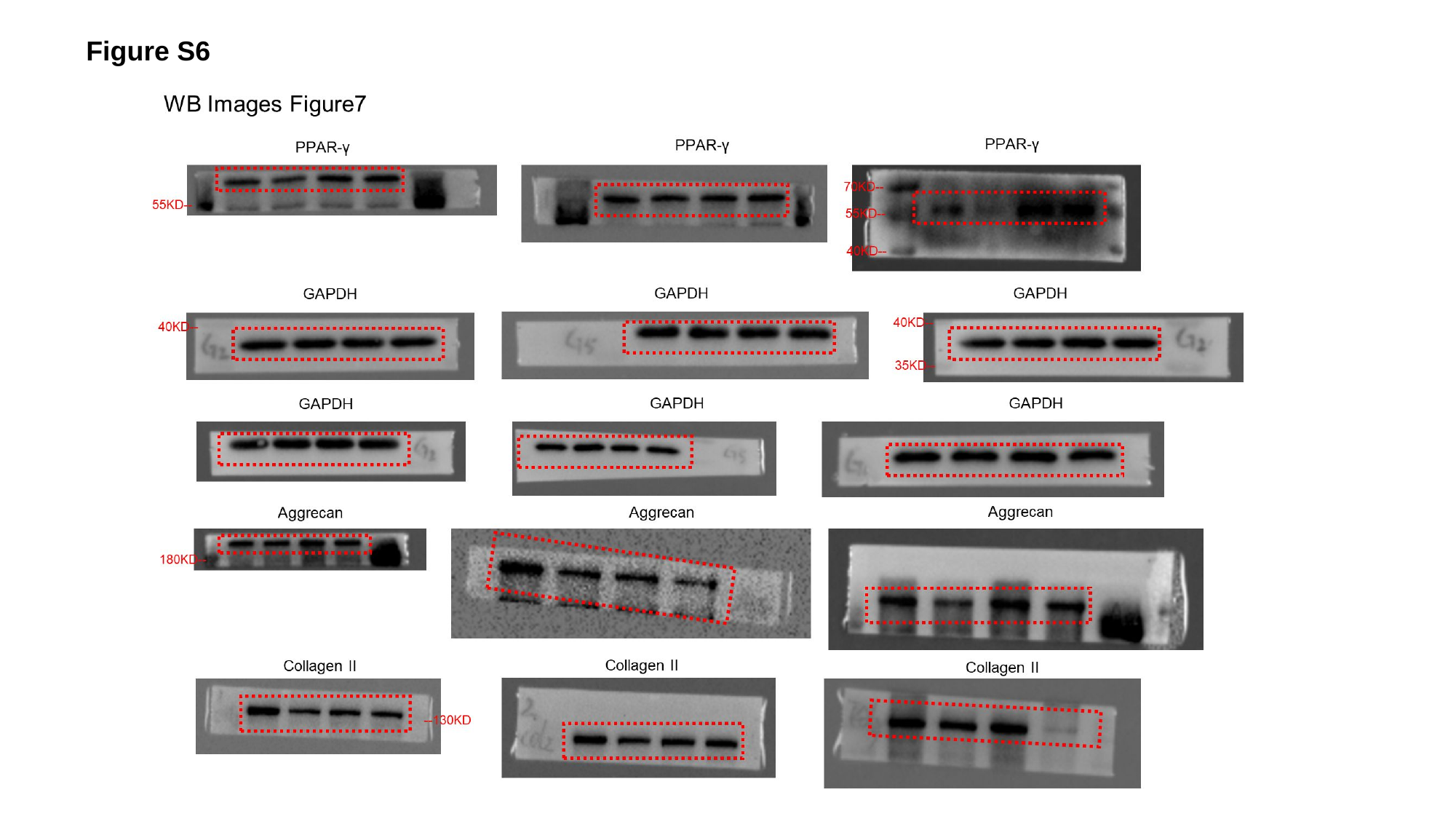

Figure S6

## Slide 7
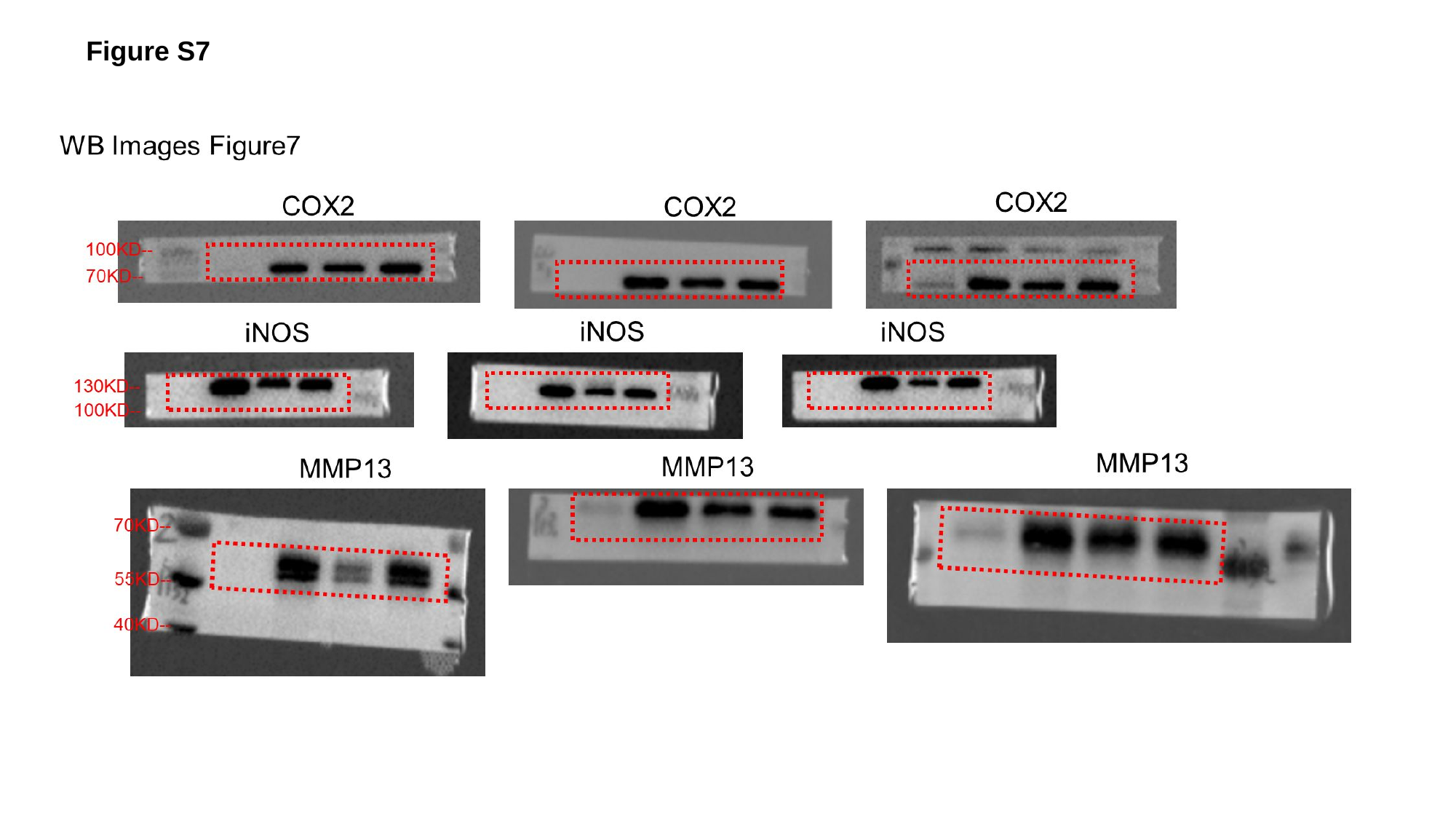

Figure S7

## Slide 8
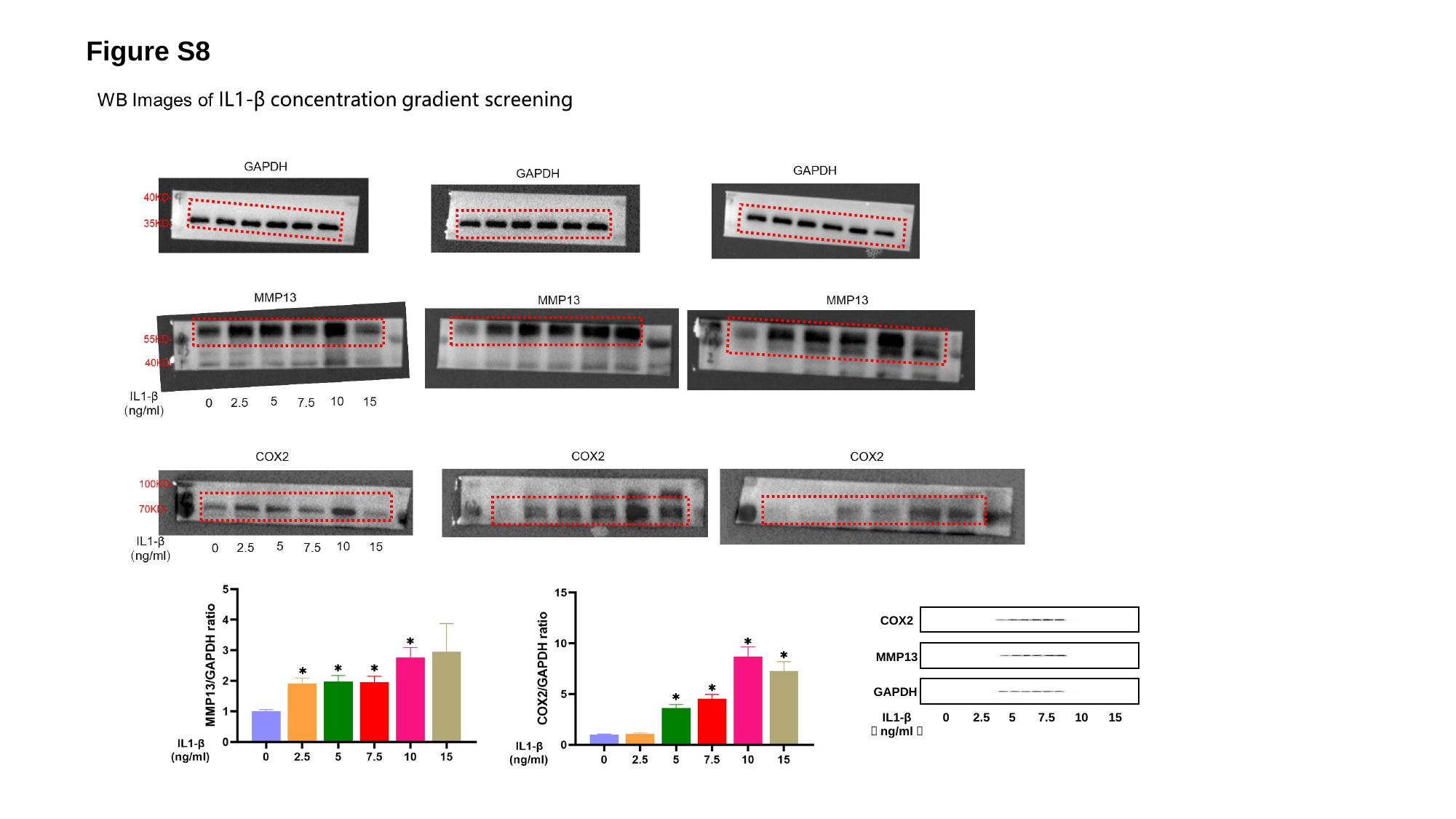

Figure S8
COX2
MMP13
GAPDH
10
15
IL1-β
（ng/ml）
0
5
7.5
2.5

## Slide 9
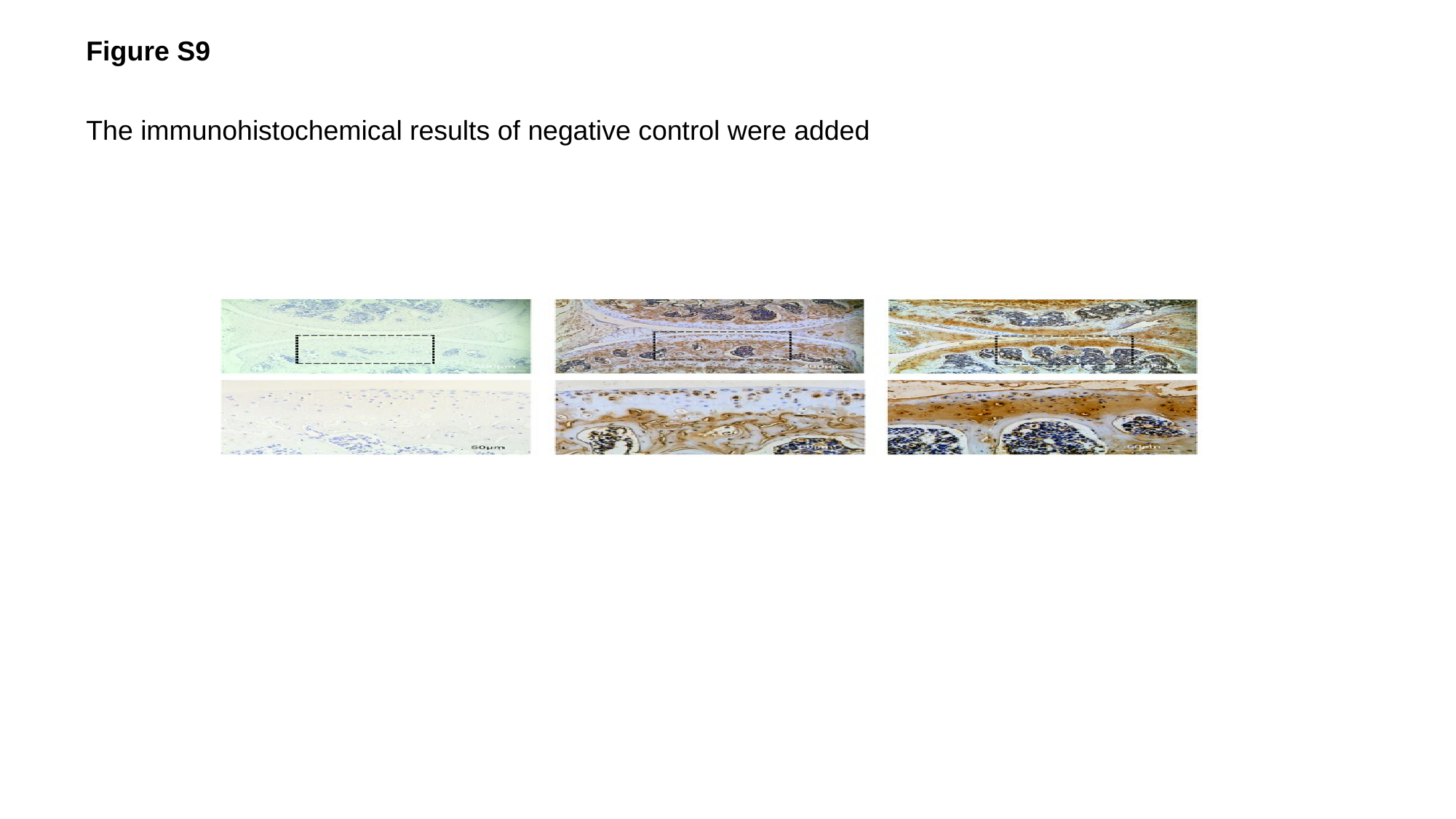

Figure S9
The immunohistochemical results of negative control were added
